# Flea infestation and molecular detection of *Rickettsia* and *Bartonella* spp. in domestic dog-associated fleas from the inter-Andean basin of Cundinamarca, Colombia

**DOI:** 10.1111/mve.70089

**Published:** 2026-06-08

**Authors:** Carlos Ramiro Silva-Ramos, Alejandro Ramírez-Hernández, Nicole L. Mendell, Laura Natalia Robayo-Sánchez, Jerson Andrés Cuéllar-Sáenz, Jesús A. Cortés-Vecino, J. Manuel Matiz-González, Marylin Hidalgo, Lucas S. Blanton, David H. Walker

**Affiliations:** 1 Grupo de Enfermedades Infecciosas, Departamento de Microbiología, Facultad de Ciencias, Pontificia Universidad Javeriana, Bogotá, Colombia; 2 Department of Pathology, University of Texas Medical Branch, Galveston, Texas, USA; 3 Grupo de Investigación Salud TI_Udistrital, Facultad de Ciencias de la Salud, Universidad Distrital Francisco José de Caldas, Bogotá, Colombia; 4 Universidad de La Salle, Bogotá D.C., Colombia; 5 Grupo de Investigación Parasitología Veterinaria, Laboratorio de Parasitología Veterinaria, Facultad de Medicina Veterinaria y de Zootecnia, Universidad Nacional de Colombia, Bogotá D.C., Colombia; 6 Molecular Genetics and Antimicrobial Resistance Unit, Universidad El Bosque, Bogotá, Colombia; 7 Department of Internal Medicine-Infectious Diseases, University of Texas Medical Branch, Galveston, Texas, USA

**Keywords:** Bartonellaceae, canines, Colombia, Rickettsiaceae, Siphonaptera, vector-borne agents

## Abstract

Dogs are common companion animals, often infested by fleas that can transmit zoonotic pathogens in rural tropical areas. In Colombia, studies on flea-borne agents in dogs are limited. Understanding the presence of these ectoparasites and associated microorganisms is crucial. To assess flea infestation and detect and identify *Rickettsia* and *Bartonella* spp. in fleas collected from dogs in Cundinamarca, Colombia. Between 2019 and 2020, fleas were collected from rural domestic dogs in 24 municipalities of western Cundinamarca. Fleas were morphologically identified and pooled for DNA extraction. Molecular screening of *Rickettsia* and *Bartonella* spp. was performed by qPCR. Positive samples underwent further characterization by conventional PCR. *Rickettsia* was additionally analyzed using RFLP, while *Bartonella* was evaluated through phylogenetic analysis. Of the 116 dogs examined across 24 municipalities, 39.7% were infested with fleas. A total of 138 fleas were collected and grouped into 46 pools, with *Ctenocephalides felis felis* identified in 94% of them. *Rickettsia* was detected in 83% of pools, with *Rickettsia asembonensis* and *Rickettsia* felis identified in most positive samples. Bartonella was found in 28% of pools; however, sequencing success was limited, and no species-level identification could be achieved. This study documents flea infestation in domestic dogs in Cundinamarca, predominantly by *C. felis felis*. High detection of *R. asembonensis* and presence of *R. felis* confirm widespread rickettsial circulation in fleas. *Bartonella* was detected less frequently, but species-level identification was not achieved.

## INTRODUCTION

Dogs are the main companion animals worldwide, largely due to the great significant mental health benefits they provide such as promoting social interaction and decreasing depression symptoms (Gee et al., 2021). In recent years, the connection between humans and dogs has strengthened, with many households considering dogs as integral members of the family nucleus (Wanser et al., 2021). However, like all living animals, dogs are also susceptible to a wide range of health issues, including infestations by ectoparasites (i.e., ticks, mites, lice, fleas), some of which can serve as vectors for several zoonotic pathogens (Colella et al., 2020; Taddesse et al., 2024).

Fleas (class Insecta, order Siphonaptera) are small, laterally flattened, wingless ectoparasites that are obligate blood feeders of a wide variety of mammalian and avian hosts (Bitam et al., 2010; Bossard et al., 2023). More than 2000 species have been officially described, but only a few of them are considered synanthropic (Bitam et al., 2010; Bossard et al., 2023). Fleas are of significant medical and veterinary importance as vectors of several pathogens, which they can transmit through regurgitation during feeding or via contaminated faeces (Eisen & Gage, 2012). While fleas tend not to exhibit strict host specificity, they often parasitize hosts that are ecologically or taxonomically similar (Krasnov et al., 2004; Sanchez et al., 2023). Some flea species are of major public health concern, such as *Tunga penetrans*, which can cause direct infestations in humans (Coates et al., 2020); while others serve as major vectors of flea-borne diseases, including *Xenopsylla cheopis*, the primary vector of *Yersinia pestis*, the etiological agent of plague, and *R. typhi* (Boyer et al., 2022); *Ctenocephalides felis felis*, commonly found on domestic dog species and capable of transmitting several pathogenic *Rickettsia* and *Bartonella* species (Moore et al., 2024); and *Pulex irritans*, which may act as a feeble vector of *Y. pestis*, *Rickettsia* and *Bartonella* species (Miarinjara et al., 2021; O’Donnell & Elston, 2020).

*Rickettsia* (order Rickettsiales, family Rickettsiaceae) is a bacterial genus composed of obligately intracellular, small, pleomorphic, Gram-negative bacteria that can be transmitted to humans and animals via ticks, lice, mites and fleas (Blanton & Walker, 2017). Flea-borne rickettsiae include the well-established pathogenic species *Rickettsia typhi* and, albeit still under debate, *Rickettsia felis* (Labruna & Walker, 2014). *Rickettsia typhi*, a member of the typhus-group (TG), is the agent of murine typhus, an acute undifferentiated febrile illness, transmitted by *X. cheopis* in an urban cycle involving rats, or by *C. felis felis* in a cycle involving opossums, with other animals such as dogs and cats acting as secondary reservoirs (Blanton, 2023; Blanton et al., 2022; Snellgrove & Goddard, 2024). *R. felis*, a suspected pathogen which belongs to the transitional group (Gillespie et al., 2007), is considered the potential agent of a disease often referred to as flea-borne spotted fever, which is associated with *C. felis felis*, but with a wide range of arthropod species harbouring it (Labruna & Walker, 2014; Minahan et al., 2023; Reif & Macaluso, 2009). In addition to these *Rickettsia* species, two additional *R. felis*-like organisms (RFLO), *Rickettsia asembonensis* and *Candidatus* R. senegalensis, have been identified in few flea species, although their pathogenicity remains unclear (Maina et al., 2019; Mediannikov et al., 2014).

*Bartonella* (order Hyphomicrobiales, family Bartonellaceae) is a bacterial genus of facultatively intracellular highly fastidious cultivable Gram-negative small rods that are transmitted by hematophagous arthropods such as flies, lice, fleas and probably ticks (Billeter et al., 2008; Saengsawang et al., 2021; Welch, 2015). To date, several *Bartonella* species have been recognized as pathogens of humans and animals, and the discovery of a great number of novel species in recent years has highlighted *Bartonella* as emerging agents, with several new species identified as pathogenic and others yet poorly understood (Iannino et al., 2018; Mira & Elitza, 2024; Mogollon-Pasapera et al., 2009). Currently, three flea-transmitted *Bartonella* species are considered pathogenic: *Bartonella henselae* (the agent of cat-scratch disease [CSD]), *B. clarridgeiae* (also associated with CSD and endocarditis) and *B. koehlerae* (a cause of culture-negative endocarditis), all primarily linked to *C. felis felis* (Avidor et al., 2004; Logan et al., 2019; Moore et al., 2024; Mosbacher et al., 2011). However, the detection of several other pathogenic *Bartonella* species in fleas underscores the importance of these arthropods as potential vectors in the transmission of *Bartonella* (Abdullah et al., 2019; Rizzo et al., 2015; Zouari et al., 2017).

In Colombia, several studies have reported flea infestations among domestic animals, including dogs, across different regions of the country (Benavides Ortiz et al., 2010; Cañón-Franco & Pérez-Bedoya, 2010). However, studies into flea-borne pathogens have been limited to few regions. In Caldas department, *R. felis* and *R. asembonensis* have been identified in fleas collected from dogs and other domestic animals (Colonia et al., 2020; Ramírez-Hernández et al., 2013); in Cauca department, these species were detected along with *Candidatus* R. senegalensis (Betancourt-Ruiz et al., 2020); and in northern Antioquia and Córdoba departments, only *R. felis* was identified (Contreras et al., 2019). In the inter-Andean basin of the Magdalena River, within Cundinamarca department, historical records have documented cases of TG rickettsioses (Cuéllar-Sáenz et al., 2023), but only a sequence suggestive of *R. asembonensis* was detected in a flea specimen collected from a man’s bed in Villeta municipality (Faccini-Martínez et al., 2016). Despite these findings, no further studies have specifically assessed the circulation of *Rickettsia* spp. or other flea-borne pathogens such as *Bartonella* spp. in fleas in this area. Due to their close association with humans, and their frequent flea infestation in rural and suburban tropical areas, dogs serve as important sentinels for flea-borne pathogens. This highlights the need to study both the extent of flea infestation and the presence of related infectious agents in canine flea populations. Thus, based on a very small sample, the aim of the present study was to assess flea infestation rates and to detect and identify *Rickettsia* and *Bartonella* species circulating in fleas collected from domestic companion dogs in multiple municipalities within the inter-Andean basin of Cundinamarca department.

## MATERIALS AND METHODS

### Study area

The inter-Andean basin of the Magdalena River is the largest intermountain basin in Colombia, which covers approximately 257,438 km^2^, representing around 24% of the national territory. This region has been heavily influenced by several anthropogenic activities, including deforestation and mining. The Magdalena River basin crosses 1540 km from south to north, through 13 departments, including Cundinamarca (https://www.basin-info.net/river-basins/magdalena-river-basin-colombia).

Cundinamarca department has a total area of 24,210 km^2^, distributed into 116 municipalities. It has a mean elevation of 3341 meters above sea level (masl), although altitudes range widely from 146 to 4151 masl. The western region of Cundinamarca forms part of the Magdalena River basin and exhibits a remarkable diversity of geological, ecological and climatic conditions. The region’s climate is mainly tropical and subtropical, which is shaped by the altimetric variations and complex relief topography of the municipalities that comprise this part of the department. It has two main alternating seasons, wet and dry, which are distributed irregularly throughout the year (https://www.cundinamarca.gov.co).

### Samples

Surveillance for flea ectoparasitosis was conducted among rural domestic companion dogs from the western part of Cundinamarca department, in the central inter-Andean region of Colombia, between 2019 and 2020, comprising six provinces: Alto Magdalena, Gualivá, Magdalena, Magdalena Central, Sabana Oeste and Tequendama, comprising 24 municipalities (Table 1). Rural homes were selected through a non-probabilistic sampling approach (snowball sampling method), primarily based on the presence of domestic companion dogs.

Fleas were manually collected directly from dogs after obtaining the owner’s consent. The owners assisted in appropriately restraining the animal during the procedure. A flea comb was used for the collection, occasionally aided by a cotton swab moistened with 70% ethanol to facilitate removal. Collected fleas were deposited in absolute ethanol to be preserved and then transported to the ‘Laboratorio de Parasitología Veterinaria’ of the ‘Facultad de Medicina Veterinaria y de Zootecnia, Universidad Nacional de Colombia, Bogotá D.C’. for taxonomic identification. Identification was performed using dichotomous taxonomic keys (Acosta & Morrone, 2003; Wall & Shearer, 2008) under an Olympus SZ2-ILST LED Illuminator Stand stereomicroscope (Spach Optics Inc., Rochester, NY, USA).

Animal sampling procedures were approved by the bioethical committee of the ‘Facultad de Medicina Veterinaria y de Zootecnia’ of the ‘Universidad Nacional de Colombia, sede Bogotá D.C.’ under approval number CB-FMVZ-UN-051-19. Flea collections were conducted under the framework permits granted to the ‘Universidad Nacional de Colombia, sede Bogotá D.C.’ by the ‘Autoridad Nacional de Licencias Ambientales (ANLA)’ by the resolution 0255 of March 14th, 2014.

### DNA extraction

DNA was extracted from flea pools made of 1–10 specimens from the same species, host and site of sampling. Flea pools were dried in a bath at 56°C for 4 h to eliminate any trace ethanol, and subsequently cut into small fragments on sterile filter paper. Then, flea pieces were macerated in 40 μL of 1× phosphate-buffered saline solution (PBS). DNA extraction was performed on macerated samples using the DNeasy^®^ Blood & Tissue Kit (250) (Qiagen, Hilden, Germany) according to the manufacturer’s instructions with the extra addition of 400 μL DNAzol^™^ Reagent (Invitrogen^™^, Life Technologies, Grand Island, NY, USA) for tissue lysis. After each extraction procedure, to assess the DNA sample integrity and rule out the presence of inhibitors, DNA was quantified spectrophotometrically using the NanoDrop 2000 (Thermo Scientific, Wilmington, DE, United States), and its quality was evaluated through a conventional PCR targeting a 710-bp fragment of the COI (mitochondrial cytochrome c oxidase subunit I) gene using the primers LCO1490 (GGTCAACAAATCATAAAGATATTGG) and HCO2198 (TAAACTTCAGGGTGACCAAAAAATCA) according to a previously reported procedure (Folmer et al., 1994). Subsequently, amplicons were visualized by electrophoresis in a 2% agarose gel stained with SYBR^™^ Safe DNA gel Stain (Invitrogen, Waltham, MA, USA).

### Detection and identification of *Rickettsia* spp.

To determine the presence of *Rickettsia* spp. DNA, a genus-specific TaqMan real-time PCR (qPCR) targeting a 147-bp fragment of the *gltA* (citrate synthase) gene was performed using the primers CS-5 (GAGAGAAAATTATATATCCAAATGTTGAT) and CS-6 (AGGGTCTTCGTGCATTTCTT), the fluorogenic probe (6-FAM-CATTGTGCCATCCAGCCTACGGT-BHQ-1) and the iQ^™^ Supermix (Bio-Rad Laboratories, Hercules, CA, USA), following the conditions suggested elsewhere (Guedes et al., 2005; Labruna et al., 2004). A positive (*Rickettsia australis* DNA) and a negative (molecular biology grade water) control were used for all reaction procedures.

All positive samples were further assessed through a duplex Taq-Man qPCR assay targeting a 266-bp fragment of the same gene using the primers gltA-F.287 (GATTTTTTAGAAGTGGCATATTTG) and gltA-R.552 (GGKATYTTAGCWATCATTCTAATAGC) and species-specific probes for *R. typhi* [CalRed610-TT(T)A(C)TA(C)A(A)AG(A)T(T)G(C)T(C) A-BHQ2] and *R. felis* [Cy5-CTA(C)GGA(G)AATT(G)CCA-BHQ3] using the iQ^™^ Supermix (Bio-Rad Laboratories, Hercules, CA, USA), following the conditions reported elsewhere (Eremeeva et al., 2008) to identify the presence of *R. typhi* and *R. felis* DNA. Two positive (*R. typhi* and *R. felis* DNA) and one negative (molecular biology grade water) controls were used for all amplification reactions.

Negative samples of the *R. typhi*/*R. felis* duplex qPCR assay were further assessed through a conventional PCR targeting a 815-bp fragment of the *sca5* (outer-membrane protein B) gene using the primers 120-2788 (AAACAATAATCAAGGTACTGT) and 120–3599 (TACTTCCGGTTACAGCAAAGT) (Roux & Raoult, 2000) with the GoTaq^®^ Green Master Mix (Promega Corporation, WI, USA) under the following conditions: initial denaturation at 95°C for 5 min; 40 cycles at 95°C for 30 s, 53°C for 30 s, and 68°C for 1 min and 30 s with final extension at 68°C for 7 min. Positive samples for the *sca5* gene were assessed through Restriction Fragment Length Polymorphism (RFLP) assay as reported elsewhere (Blanton, Quade, & Bouyer, 2019) to identify other flea-borne *Rickettsia* species. Briefly, an enzymatic digestion was performed on amplified products of *sca5* using the *NlaIV* (New England BioLabs, Ipswitch, MA, USA) restriction enzyme, by incubating 5 μL of the obtained amplicon with 3 μL of molecular grade water, 1 μL of the restriction enzyme and 1 μL of manufacturer-selected enzyme buffer, overnight at 37°C water bath. Then, digested products were evaluated by electrophoresis in a 4%–12% NOVEX TBE polyacrylamide gel (Thermo Fisher Scientific, Waltham, MA, USA) at 200 V for 30 min, stained with ethidium bromide, and visualized with a UV transilluminator.

### Detection and identification of *Bartonella* spp.

To determine the presence of *Bartonella* DNA, a genus-specific qPCR assay was performed targeting a 301-bp fragment of the ssrA (transfer-mRNA) gene using the primers ssrA-F (GCTATGGTAATAAATGGACAATGAA ATAA) and ssrA-R (GCTTCTGTTGCCAGGTG), together with a fluorogenic probe (5′-FAM-ACCCCGCTTAAACCTGCGACG-3′-BHQ1) and the iQ^™^ Supermix (Bio-Rad Laboratories, Hercules, CA, USA), following the conditions reported elsewhere (Diaz et al., 2012). A positive (*B. henselae* DNA) and a negative (molecular biology grade water) control were used for all reactions.

Positive samples were further screened by PCR targeting two additional complementary genes: a 379-bp fragment of the *gltA* gene using the primers BhCS.781p (GGGGACCAGCTCATGGTGG) and BhCS.1137n (AATGCAAAAAGAACAGTAAACA), and a 333-bp fragment of the rpoB (RNA polymerase β subunit) gene employing the primers rpoBF (GCACGATTYGCATCATCATTTTCC) and rpoBR (CGCATTATGGTCGTATTTGTCC), following the PCR conditions reported elsewhere (Norman et al., 1995; Paziewska et al., 2011) and with the same positive and negative controls of the *ssrA* qPCR protocol. All PCR amplified products were evaluated by electrophoresis in a 2% agarose gel stained with ethidium bromide. All obtained amplicons were bidirectionally sequenced using the Sanger method.

All chromatograms of both forward and reverse sequences retrieved were checked to trim low-quality regions, and then both forward and reverse sequences were assembled to obtain a consensus sequence using the SnapGene^®^ Viewer 6.0.5 software and subsequently compared with reported sequences from the NCBI GenBank database using the BLASTn server (Ye et al., 2006). Reference sequences of *gltA* and *rpoB* for all recognized *Bartonella* species were retrieved from the NCBI nucleotide sequence repository. Goodquality sequences obtained in the present study were aligned together with retrieved *Bartonella* reference sequences using the ClustalW algorithm (Thompson et al., 1994). An individual phylogeny was elaborated for each targetted marker. For this, the alignment matrices for each marker were used to build a maximum likelihood (ML) tree. The ML tree was elaborated using a GTR + GAMMA model, which was identified as the best evolution model according to the Bayesian information criterion, with a bootstrap branch support analysis with 1000 replicates. All alignment matrices and phylogenetic analyses were constructed using the MEGA X software version 10.0.5 (Kumar et al., 2018).

## RESULTS

### Flea infestation

A total of 116 domestic companion dogs were surveyed across 24 municipalities of Cundinamarca department. The number of dogs surveyed per municipality ranged from 1 to 12, with a mean of 4.8, a median of 5, and a mode of 5. Flea infestation among these animals was observed in 39.7% (46/116) of individuals. Among municipalities with at least seven dogs surveyed, the highest flea infestation rate was found in Anolaima municipality (78% [7/9]). No records of flea infestation among dogs were found in five of the surveyed municipalities (Table 1).

**FIGURE 1:**
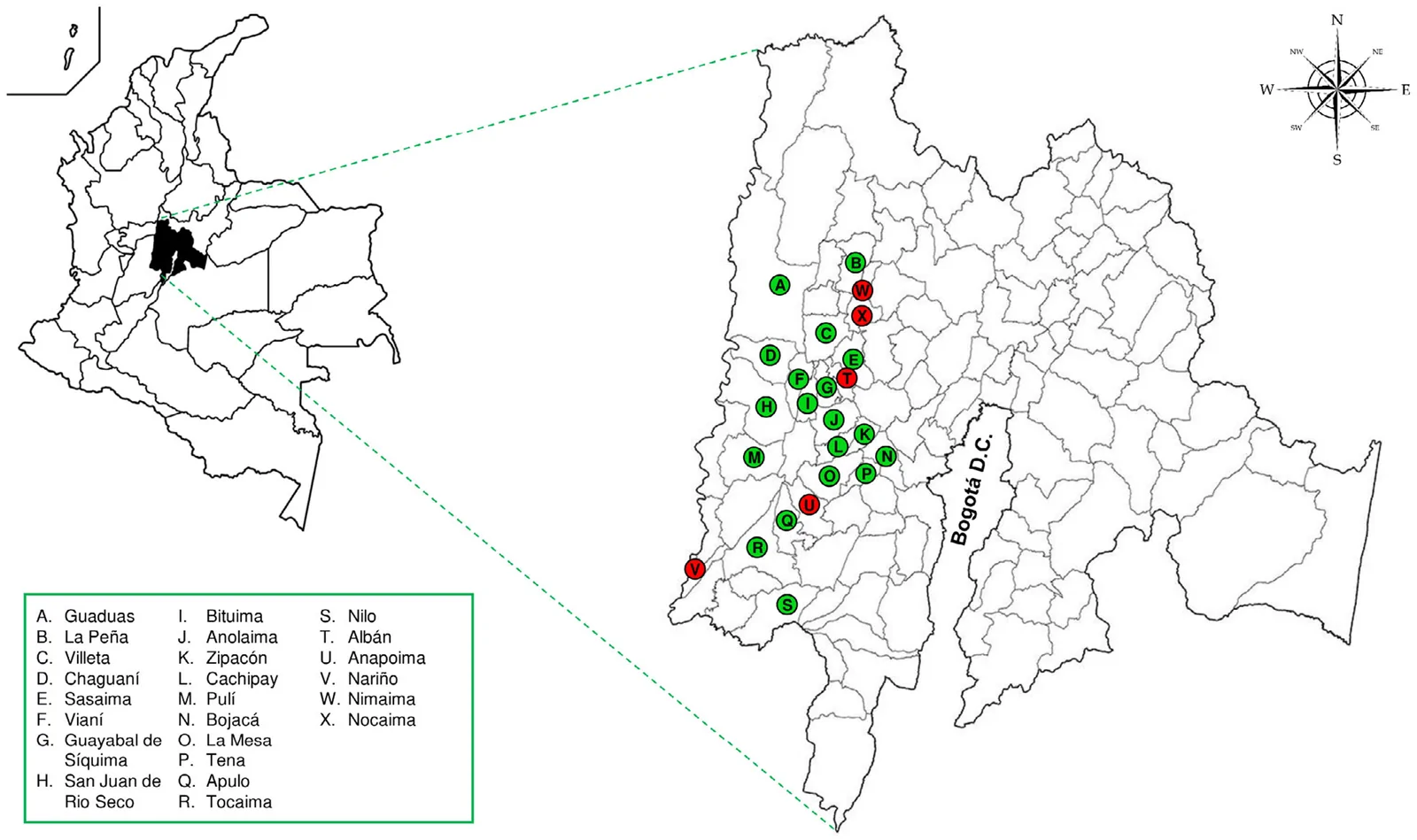
Map of Cundinamarca where flea infestation among domestic dogs was observed. Green circles with a letter inside represent each municipality where flea-infested dogs were found. Red circles with a letter inside represent municipalities where dog sampling was performed but no evidence of ectoparasites were observed. All the municipalities are described in the figure according to each letter.

From all 46 domestic companion dogs found infested across 19 municipalities (Figure 1), a total of 138 fleas were collected, which were grouped into 46 different pools. Each pool contained between one and 10 flea specimens. Based on taxonomic keys, 94% (43/46) of the flea pools were identified as *C. felis felis*. The remaining three pools were identified as *Ctenocephalides* canis (*n* = 1), *P. irritans* (*n* = 1), and one pool that could not be identified to species level because of external damage and was thus classified as *Ctenocephalides* sp. Regarding the geographic origin of the samples, more than half of the fleas collected were collected from Anolaima (17.4% [24/138]), Guayabal de Síquima (12.3% [17/138]), La Mesa, San Juan de Rioseco and Vianí (10.9% [15/138] each) municipalities. More information can be observed in Table 2.

### Detection and identification of *Rickettsia* spp.

The COI gene was successfully amplified from all DNA-extracted flea samples as expected. Of these, 83% (38/46) were positive for the rickettsial *gltA* gene, with cycle threshold (Ct) values ranging between 17.43 and 33.77. Among the positive flea pools, 90% (34/38) were identified as *R. asembonensis*, while 8% (3/38) were identified as *R. felis*. In one pool, amplification of the *sca5* gene was unsuccessful, thus it was not possible to identify the detected *Rickettsia* species. All positive pools corresponded to *C. felis felis*, whereas pools of *C canis, P. irritans* and *Ctenocephalides* sp. tested negative. Positive flea pools were distributed across 16 of the 19 municipalities where fleas were collected. Further details regarding the origin of the positive samples are presented in Table 2. And more specific details such as sampling date coordinates, and altitude can be observed in Table S1 (Silva-Ramos, 2026).

### Detection and identification of *Bartonella* spp.

**FIGURE 2:**
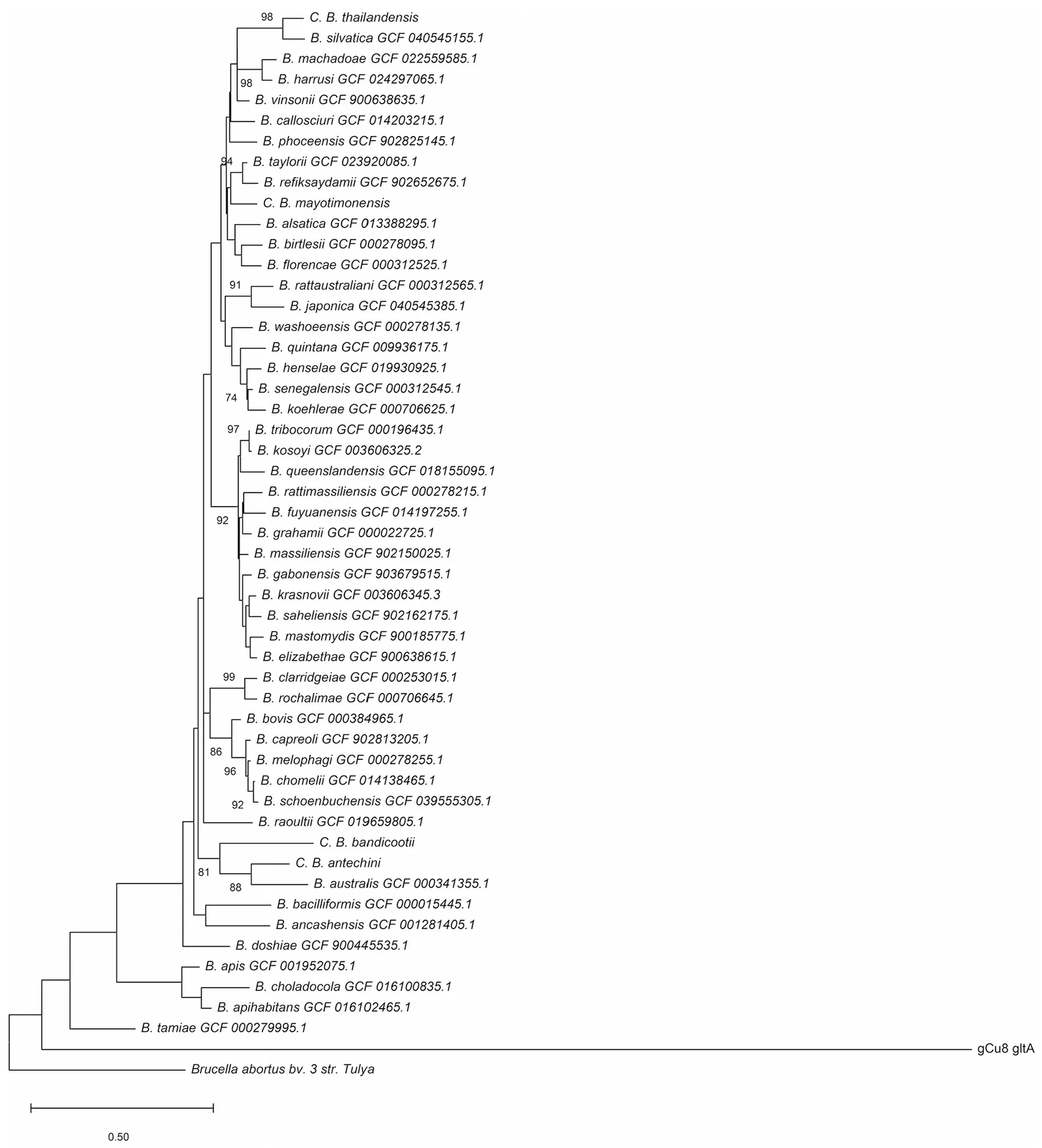
Maximum likelihood phylogenetic tree of *Bartonella gltA* sequences. The sequence retrieved in this study is labelled gCu8 gltA. Bootstrap values >70% are shown at nodes.

The presence of *Bartonella* DNA was evidenced in 28% (13/46) of all the flea pools collected, with Ct values ranging from 34.97 to 42.16. Positive flea pools were distributed across only eight of the 19 municipalities where fleas were collected (Tables 2 and S1) (Silva-Ramos, 2026). From these samples, the *Bartonella* rpoB gene was amplified in 12 samples and the *gltA* was amplified in nine samples. At least one of the two complementary genes was amplified in each positive pool. Attempts were made to sequence all of the amplicons; however, only one *gltA* good-quality sequence was obtained. BLASTn analysis revealed 100% identity and 98% query coverage with a previously reported *Bartonella* sequence (GenBank accession no. GU056187.1) obtained from the blood of a dog in central Taiwan (Tsai et al., 2011), and 99.2% identity and 98% query coverage with a *Bartonella* sequence (GenBank accession no. KP126473.1) from a flea in Tunisia (Zouari et al., 2017). However, phylogenetic analysis using reference sequences from all currently recognized *Bartonella* species and selected *Candidatus* taxa showed that the retrieved sequence (gCu8 gltA) did not cluster with any known species, instead forming a highly divergent branch (Figure 2) making species-level identification not possible.

## DISCUSSION

Despite the recognized role of fleas as vectors of several zoonotic pathogens, few studies in Colombia have specifically focused on the detection of flea-borne agents in specimens collected from companion animals such as dogs and cats (Betancourt-Ruiz et al., 2020; Colonia et al., 2020; Contreras et al., 2019; Ramírez-Hernández et al., 2013). This represents a significant gap in understanding the dynamics of flea-related risks in the context of close human-animal interactions, particularly in rural and peri-urban areas where flea infestation may be more prevalent and vector control measures are often limited. Beyond their zoonotic potential, fleas can directly compromise animal health by causing allergic dermatitis, anaemia and transmitting pathogens of veterinary importance (Linardi, 2017). Given the close contact between companion animals and humans, particularly in domestic settings, ongoing monitoring of ectoparasite infestations and the pathogens they harbour is essential to safeguard both animal and human health (Joosten et al., 2020). The present study contributes to addressing this knowledge gap by providing updated data on the occurrence and identification of flea ectoparasites associated with household dogs in central Colombia, while also revealing the presence of flea-borne *Rickettsia* and *Bartonella* species within these vectors.

Flea infestation in domestic dogs was common in the study area, highlighting the importance of proper dog care by owners in these regions to prevent flea-related issues both in dogs and their owners (Iannino et al., 2018; Linardi, 2017). This pattern may reflect environmental conditions that favour flea development in rural areas where regular ectoparasite control in rural dogs may be limited. The higher flea abundance observed in some municipalities may also reflect local ecological conditions, host density and differences in dog management practices. The cat flea, *C. felis felis*, was identified as the predominant species. This flea species is widely recognized as the most common flea ectoparasite of domestic cats and dogs globally, particularly in tropical and subtropical regions (Moore et al., 2024; Tay, 2013). Although *C. felis felis* has been recognized as a cause of flea-allergy dermatitis (Youssefi et al., 2014), its importance as a vector of several zoonotic pathogens, such as *B. henselae* and *Rickettsia typhi* (Blanton, Vohra, et al., 2019; Duscher et al., 2018), remains underappreciated. The high rate of *C. felis felis* observed in this and previous studies (Betancourt-Ruiz et al., 2020; Ramírez-Hernández et al., 2013) reinforces the urgent need for improved ectoparasite control strategies in Colombia, mainly in rural areas where resources may be limited. In addition, the detection of *C. canis* and *P. irritans*, although less prevalent, is also relevant since these species can also contribute to the transmission of zoonoses in certain regions (Ratovonjato et al., 2014; Venzal et al., 2006).

The detection of *Rickettsia* in flea pools, predominantly *R. asembonensis* and to a lesser extent, *R. felis*, indicates frequent circulation of these agents in fleas infesting domestic dogs in western Cundinamarca. Similar patterns have been reported in fleas from companion animals in other Latin American countries such as Brazil, Guatemala and Costa Rica (Silva et al., 2017; Troyo et al., 2012). In Colombia, only limited data exist from Caldas department, which lies geographically close to the present study area, and where a similar diversity of *Rickettsia* species has been identified (Colonia et al., 2020). Notably, Caldas department, a region where murine typhus was historically reported (Hidalgo et al., 2008), lies within the Magdalena River inter-Andean basin, and is geographically close to the area where the present study was performed; however, only *R. asembonensis* and *R. felis* were identified. Although recent studies have attempted to associate *R. asembonensis* with cases of acute undifferentiated febrile illness (Loyola et al., 2024), its widespread distribution without documented cases of human and animal disease suggests that it may simply be part of the microbiome of different arthropod hosts (Maina et al., 2019). *R. felis*, in contrast, has been proposed as an emerging agent related to flea-borne spotted fever (Brown & Macaluso, 2016); however, its pathogenic role remains uncertain, as current evidence is inconsistent with traditional paradigms of rickettsial diseases (Labruna & Walker, 2014). To date, there are no isolates from ill persons and there are no convincing serologic data to support *R. felis* is pathogenic. The high infection rate of both species in fleas highlights the need for further research into their potential roles within the flea microbiome, including their possible interference with and modulation of other flea-borne pathogens, both rickettsial and non-rickettsial such as *Y. pestis*, to better understand their biological significance (Blanton & Walker, 2017; Maina et al., 2019).

*Bartonella* was detected less frequently than *Rickettsia*, suggesting it may be a less common microorganism in fleas in this region. Similar observations have been reported in nearby countries, including Costa Rica, Mexico and Peru (Rizzo et al., 2015; Rojas et al., 2015; Tobar et al., 2020), suggesting that *Bartonella* may be a less common microorganism in fleas. In Colombia, studies on *Bartonella* are still scarce, this being the first report of these bacteria in fleas collected from companion dogs, and only a previous similar study documenting *Bartonella rochalimae* and *B. vinsonii* subsp. *berkhoffii* in stray dogs from Bogotá (Brenner et al., 2013). Although two additional genetic markers were targeted in the present study (Norman et al., 1995; Paziewska et al., 2011), only one good-quality sequence was recovered. This sequence showed high divergence and did not cluster with any recognized species. Similar findings have been documented worldwide, where *Bartonella* detected from fleas could not be identified and fully characterized (Rojas et al., 2015; Zouari et al., 2017), reinforcing the emerging status of this genus as novel species continue to be described from several vertebrate and invertebrate hosts (Çelebi et al., 2025; Liu et al., 2022). Some of the retrieved sequences matched *Wolbachia* spp., an endosymbiont commonly found in fleas (Flatau et al., 2025), and were therefore excluded from further analysis. This highlights the importance of careful primer selection in future studies, and reinforces the necessity of sequencing to confirm accurate identification of *Bartonella*. Further amplification of additional targets was not possible due to limited DNA availability. Future studies are needed to better define the actual species circulating in fleas in the region and in the country, given the increasing relevance of *Bartonella* to both human and veterinary health (Breitschwerdt, 2014).

As with any field-based investigation, this study faced several limitations that should be acknowledged. In some municipalities, the low number of dogs surveyed may have affected the representativeness of both flea infestation and infection rates with flea-borne bacterial agents. Moreover, limited sequencing success, especially for *Bartonella* genes, hindered deeper genetic characterization. Nonetheless, the findings presented here offer valuable baseline information on flea infestation in domestic dogs and the presence of *Rickettsia* and *Bartonella* spp. in its fleas from a region of Colombia where such data were previously unavailable. Further studies on fleas from wildlife hosts, mainly opossums and rodents, are needed. Importantly, the molecular detection of these bacteria in fleas indicates their environmental circulation but does not necessarily imply active transmission to humans or the occurrence of human infection. Additional epidemiological and clinical investigations would be required to assess the actual zoonotic risk.

## CONCLUSION

This study provides data on flea infestation among domestic companion dogs and the presence of *Rickettsia* and *Bartonella* in fleas collected from these animals in the western region of Cundinamarca department, Colombia. A substantial proportion of surveyed dogs were infested with fleas, mostly *C. felis felis*, emphasizing the need for ectoparasite control measures in companion animals, particularly in rural and peri-urban areas. The high frequency of *Rickettsia* detection, mainly *R. asembonensis*, and the identification of *R. felis* in a smaller number of pools reflect the widespread presence of these rickettsial agents in flea populations, though their current importance in human and animal health remains to be clarified. *Bartonella* was detected in fewer samples, and while limited sequence data were obtained, preliminary analysis suggests genetic divergence that warrants further investigation. These findings highlight the relevance of continued molecular surveillance of flea-borne pathogens and the need for integrated human–animal health approaches in understudied regions of Colombia.

## Supplementary Material

supplement Table S1. Samples data and results obtained for each sample.

Additional supporting information can be found online in the Supporting Information section at the end of this article.

## Figures and Tables

**TABLE 1:** Flea infestation rate in domestic companion dogs across municipalities surveyed in Cundinamarca, Colombia.

Municipality	Number of dogs surveyed	Number of dogs infested with fleas (%)
Albán	1	0 (0)
Anapoima	5	0 (0)
Anolaima	9	7 (78)
Apulo	9	2 (22)
Bituima	1	1 (100)
Bojacá	3	3 (100)
Cachipay	2	1 (50)
Chaguaní	3	1 (33)
Guaduas	5	4 (80)
Guayabal de Síquima	4	3 (75)
La Mesa	12	5 (42)
La Peña	8	2 (25)
Nariño	2	0 (0)
Nilo	7	2 (29)
Nimaima	2	0 (0)
Nocaima	7	0 (0)
Pulí	3	1 (33)
San Juan de	5	4 (80)
Rioseco		
Sasaima	2	1 (50)
Tena	4	1 (25)
Tocaima	4	1 (25)
Vianí	5	4 (80)
Villeta	12	2 (17)
Zipacón	1	1 (100)
All municipalities	116	46 (39.7)

**TABLE 2:** Flea species collected from dogs and detection of *Rickettsia* spp. and *Bartonella* spp. in flea pools from selected municipalities of Cundinamarca department, Colombia.

Municipality	Number of fleas collected	Number of flea pools	Flea species identified	*Rickettsia* spp. detection (%)^a^	*Rickettsia* spp. identified	*Bartonella* spp. detection (%)^b^
Anolaima	24	7	*C. felis*	7 (100)	*R. asembonensis, R. felis*	1 (14)
Apulo	4	2	*C. felis*	2 (100)	*R. asembonensis*	1 (50)
Bituima	3	1	*C. felis*	1 (100)	*R. asembonensis*	0 (0)
Bojacá	4	3	*C. canis, C. felis*	1 (33)	*R. asembonensis*	0 (0)
Cachipay	6	1	*C. felis*	1 (100)	*R. asembonensis*	0 (0)
Chaguaní	2	1	*C. felis*	1 (100)	*R. asembonensis*	1 (100)
Guaduas	6	4	*C. felis*	4 (100)	*R. asembonensis*	4 (100)
Guayabal de Síquima	17	3	*C. felis*	3 (100)	*R. asembonensis*	2 (67)
La Mesa	15	5	*C. felis*	5 (100)	*R. asembonensis*	2 (40)
La Peña	4	2	*C. felis*	2 (100)	*R. asembonensis*	1 (50)
Nilo	4	2	*C. felis*	0 (0)		0 (0)
Pulí	4	1	*C. felis*	1 (100)	*R. asembonensis*	0 (0)
San Juan de Rioseco	15	4	*C. felis, P. irritans*	3 (75)	*R. asembonensis*	0 (0)
Sasaima	2	1	*C. felis*	1 (100)	*R. asembonensis*	0 (0)
Tena	1	1	*Ctenocephalides* sp.	0 (0)		0 (0)
Tocaíma	4	1	*C. felis*	0 (0)		0 (0)
Vianí	15	4	*C. felis*	4 (100)	*R. asembonensis, R. felis*	1 (25)
Villeta	6	2	*C. felis*	1 (50)	*R. asembonensis*	0 (0)
Zipacón	2	1	*C. felis*	1 (100)	*R. felis*	0 (0)
All municipalities	138	46		38 (83)		13 (28)

a The frequency was determined from the results obtained by *gltA* qPCR over the number of flea pools.

b The frequency was determined from the results obtained by *ssrA* qPCR over the number of flea pools.

## Data Availability

The data supporting this study are available at DOI: https://doi.org/10.5281/zenodo.19069330.
